# Intestinal Reconstruction in Infants Under Epidural Anesthesia Without Invasive Airway: A Prospective Case Study

**DOI:** 10.3390/jcm14175943

**Published:** 2025-08-22

**Authors:** Daniela Marhofer, Markus Zadrazil, Philipp L. Opfermann, Caspar Wiener, Peter Marhofer, Werner Schmid

**Affiliations:** 1 Department of Anesthesia, Critical Care and Pain Medicine, Medical University of Vienna, Waehringer Guertel 18–20, 1090 Vienna, Austria; daniela.marhofer@meduniwien.ac.at (D.M.); philipp.opfermann@meduniwien.ac.at (P.L.O.); peter.marhofer@meduniwien.ac.at (P.M.); 2 Department of Pediatric Surgery, Division of Pediatric Surgery, Medical University of Vienna, 1090 Vienna, Austria; caspar.wiener@meduniwien.ac.at; 3 Department of Special Anesthesia and Pain Therapy, Medical University of Vienna, 1090 Vienna, Austria; werner.schmid@meduniwien.ac.at

**Keywords:** general anesthesia, pediatrics, ultrasonography, intestinal reconstruction, epidural anesthesia

## Abstract

**Background and Aims**: This study explored the feasibility of performing intestinal reconstruction after enterostomy in infants using ultrasound-guided epidural anesthesia with sedation, aiming to avoid invasive airway manipulation and the use of opioids. **Methods**: We included twenty infants scheduled for intestinal reconstruction in this prospective case series. Success was defined by the absence of additional general anesthesia and invasive airway management. The secondary endpoints were the need for additional intraoperative anesthetic and analgesic drugs and postoperative analgesics in the recovery room. The study was approved by the Ethics Commission at the Medical University of Vienna (ref. 1133/2017, approval date 24 August 2017) and registered in the German Clinical Trial Register (DRKS ID: DRKS00012683, approval date 15 July 2019). **Results**: Nineteen out of twenty procedures were successfully performed with epidural anesthesia under spontaneous breathing and without airway manipulation; one child required endotracheal intubation due to an unexpected, extensive surgical procedure. No child needed systemic analgesics in the recovery room. **Conclusions**: Epidural anesthesia with sedation can effectively minimize airway manipulation and reduce general anesthesia requirements for intestinal reconstruction in infants.

## 1. Introduction

Intestinal reconstruction in neonates and babies is the final step in surgical treatment after alimentary tract pathologies such as necrotizing enterocolitis, atresia, stricture, perforation, and anal atresia [1]. Post-stoma complications can include growth impairment and infections, making stoma closure and intestinal reconstruction mandatory in the smallest patient category [2].

General anesthesia (GA) with tracheal intubation is standard for pediatric abdominal surgeries but can lead to respiratory incidents, prolonged mechanical ventilation, and potential neurodevelopmental issues due to various GA drugs [3]. Previous studies have shown that epidural anesthesia is an effective alternative to GA with endotracheal intubation for a large variety of surgical procedures, including open pyloromyotomy [4], open transvesical ureteric reimplantation [5], or subumbilical laparoscopic procedures [6]. Thus, epidural anesthesia, potentially minimizing GA drug use, could also be effective for intestinal reconstruction in young children.

We, therefore, conducted an observational study in 20 children undergoing intestinal reconstruction after various gastrointestinal pathologies using ultrasound-guided single-shot epidural anesthesia with light sedation and avoidance of an instrumented airway. Success was defined as the absence of invasive airway manipulation and intraoperative opioid administration.

## 2. Materials and Methods

### 2.1. Preparations, Enrolling Patients, and Exclusion Criteria

**Figure 1:**
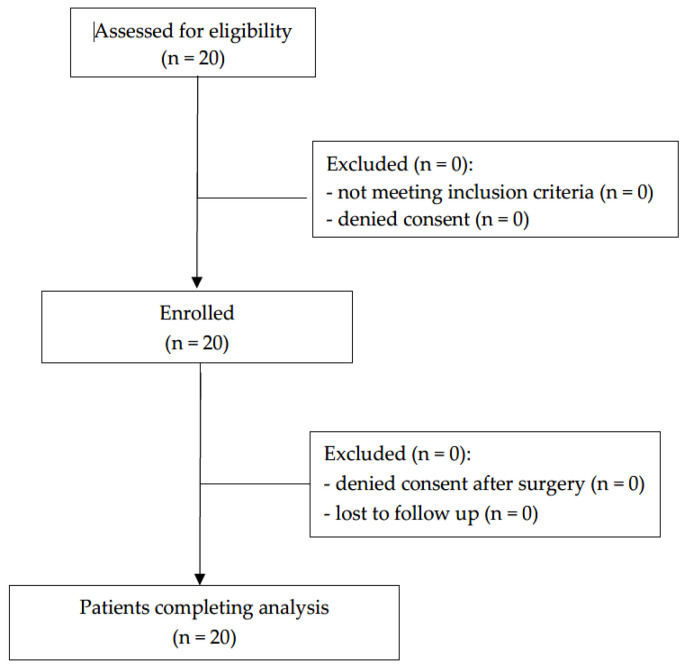
STROBE diagram of study population.

The Ethics Commission at the Medical University of Vienna approved this prospective case series (ref. 1133/2017, approval date 24 August 2017). It was registered in the German Clinical Trial Register (DRKS ID: DRKS00012683, approval date 15 July 2019), and the study design adhered to the principles outlined in the STROBE statement (Figure 1). The study execution followed applicable regulations and guidelines. Written consent was obtained from parents or legal guardians of all the participating children, who were informed about the study’s nature, scope, and procedures.

Twenty neonates and infants scheduled for stoma closure and intestinal reconstruction underwent an eligibility assessment and were enrolled at our hospital, Division of Pediatric Surgery, Medical University of Vienna, Austria, from May 2018 to August 2021. The exclusion criteria included allergies to local anesthetic drugs, local infection at the intended site, coagulation disorders, or thrombocytopenia. Additionally, participants who had taken part in another clinical study within the preceding 4 weeks before surgery, those with clinically relevant ECG abnormalities such as AV-block or bradycardia, or parents/legal guardians unable to comprehend the study protocol and associated procedures were excluded. The surgical exclusion criteria were not explicitly specified.

### 2.2. Anesthesia Management

The standard care protocol in our department during the time of the study mandated a preoperative fasting period of 6 h for solid food, 4 h for breast milk, and 2 h for clear fluids. Premedication was not performed, since babies <6 months of age are generally not premedicated in our department. Cardiorespiratory monitoring, including ECG, non-invasive arterial pressure, and SpO_2_, commenced with the child positioned on a forced-air warming blanket (Bair Hugger; Arizant, Eden Prairie, MN, USA). Sedation was started via a face mask delivering a combination of sevoflurane 8 Vol% and oxygen/air (FiO_2_ 50%). Thereafter a vascular access was established and a bolus of 1–2 mg kg^−1^ propofol was administered, followed by a continuous propofol infusion of 5 mg kg^−1^ h^−1^ and fluid administration with Elo-Paed balanced 10 mL kg^−1^ h^−1^ (Fresenius Kabi, Graz, Austria) [7]. Per study protocol, spontaneous respiration should be maintained throughout the entire perioperative period. During surgery, continuous confirmation of spontaneous breathing was ensured using an end-tidal CO_2_ line connected to a face mask secured with adhesive tape, through which oxygen-enriched air (FiO_2_ 0.40) was delivered.

### 2.3. Ultrasound-Guided Neuraxial Procedures

We performed ultrasound-guided caudal anesthesia for infraumbilical surgery and epidural anesthesia at the thoracolumbar transition (TH12/L1) for supraumbilical surgery. Both techniques were performed in accordance with our previous publications. All infants were placed in a left lateral position with knees flexed. Using an M-Turbo (SonoSite) ultrasound device with a linear transducer and a sterile probe cover, we visualized the respective neuraxial structures. For caudal punctures, we used the ‘immobile needle technique’ with a short-bevel 24G canula and a prefilled injection line, injecting 1.0 mL kg^−1^ ropivacaine 3.8 mg ml^−1^ into the caudal space [8]. Epidural blockade at Th12-L1 was performed using a 20G Tuohy canula and a loss-of-resistance syringe. After placing the canula via the ‘loss-of-resistance technique’, we injected 0.5 mL kg^−1^ ropivacaine 3.8 mg ml^−1^ into the epidural space under ultrasound guidance [9]. The rationale for using ropivacaine with a concentration of 3.8 mg ml^−1^ is based on our 20 years’ experience of using ropivacaine for epidural anesthesia, where we detected a profound sensory blockade without any cardiovascular or central neurological side effects, which is also well described in the literature [10].

### 2.4. Assessment of Anesthesia and Emergency Procedures

**Figure 2:**
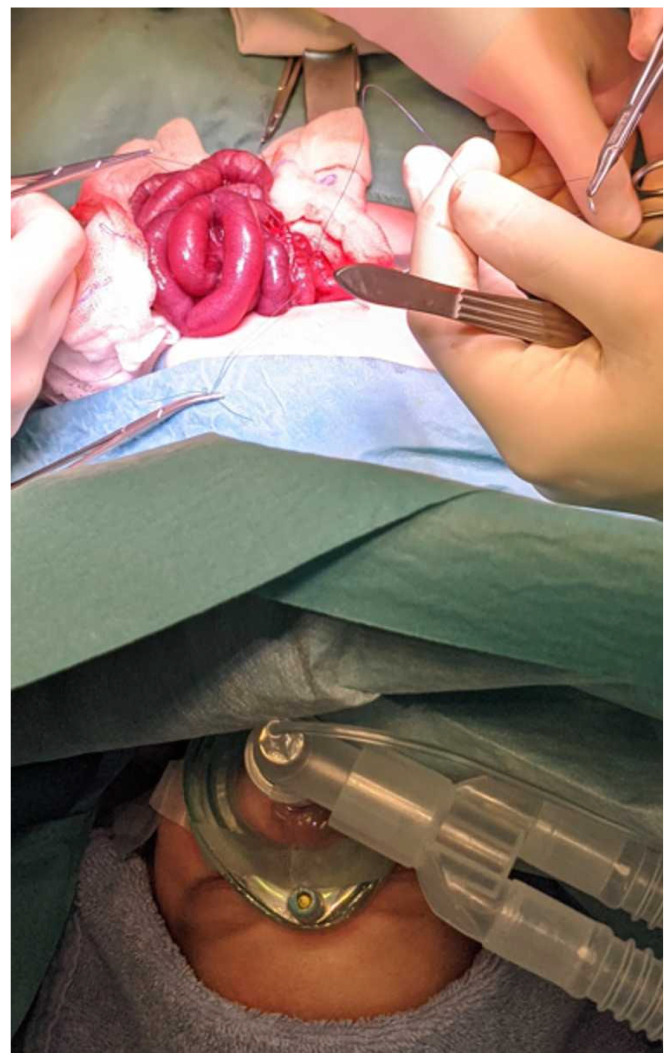
Intraoperative setting, where the child underwent the surgical procedure via epidural anesthesia, sedation, and spontaneous breathing without invasive airway management.

Ten minutes after performing the blockade, surgeons conducted Pinprick testing by applying stimulation with forceps to the area of the surgical incision. The prerequisites for subsequent surgical incision were unaltered hemodynamic parameters (an increase in heart rate < 15% from initial values, an increase in blood pressure < 15% from initial values) and no movement of the infants. Any resultant movement or an increase in heart rate or blood pressure by ≥ 15% from baseline was considered an inadequate blockade. Therefore, a successful blockade was defined if the entire surgical procedure could be performed under epidural blockade with sedation (as described above, Figure 2).

A protocol of sequential management was used for potential perioperative adverse events. Any respiratory depression (brady- or apnea) initiated careful bag-mask ventilation with <10 mmHg of inspiratory pressure. In cases of prolonged respiratory depression, endotracheal intubation was performed in accordance with our departmental standard (propofol 4 mg kg^−1^, rocuronium 0.6 mg kg^−1^, and fentanyl 0.005 mg kg^−1^). Bradycardia or hypotension to >25% below baseline was treated with atropine 0.02 mg kg^−1^ or a fluid bolus of 10 mL kg^−1^, respectively.

### 2.5. Postoperative Management in the Recovery Room

Following transfer to the post-anesthesia care unit, the pain status of the children was monitored using the Face, Legs, Activity, Cry, and Consolability (FLACC) score [11]. The FLACC score assesses objective behavioral variables such as crying, facial expression, posture of the torso and legs, and motor restlessness. Each variable is rated on a three-point scale (0 = none, 1 = moderate, and 2 = severe), with a maximum cumulative score of 10. The scores were evaluated upon admission to the recovery room and at 30 min intervals during the postoperative period until the neuraxial anesthesia resolved. If the FLACC score was ≥ 4 in two consecutive assessments, the child received an intravenous dose of nalbuphine 0.1 mg kg^−1^. A clinical examination for local infections at the puncture site was conducted, and the use of systemic analgesics was reviewed 24 h post-surgery.

### 2.6. Study Endpoints and Data Analysis

The primary endpoint of the investigation was achieving successful blockade, defined as intraoperative anesthesia management via epidural blockade and light sedation in spontaneous breathing without airway instrumentation. Secondary endpoints were defined as the use of fentanyl/additional propofol during surgery and the administration of postoperative analgesics in the recovery room. The collected data were analyzed using spreadsheets (Excel 2016; Microsoft, Redmond, WA, USA) and statistical software (Prism 10.4.1 (627); GraphPad Software Inc., San Diego, CA, USA), with the normal distribution assessed using D’Agostino–Pearson testing.

## 3. Results

This study included twenty children scheduled for intestinal reconstruction. Patient flow and relevant data are presented in Figure 1 and Table 1, respectively. Initial surgical indications are illustrated in Table 2.

The primary endpoint was achieved in 95% (*n* = 19) of the cases, where intraoperative anesthetic management was successfully performed via epidural blockade without airway instrumentation. Caudal blockade was performed in 35% (*n* = 7), and epidural anesthesia at the thoracolumbar transition was performed in 65% (*n* = 13) of cases. One infant (age: 65 days, weight: 3.9 kg) required additional analgesia (20 µg fentanyl during the intraoperative period) and subsequent endotracheal intubation due to insufficient epidural blockade during an extensive adhesiolysis procedure. The duration of this specific surgical procedure (right hemicolectomy with ileum to transverse colon anastomosis) was 152 min. The infant was extubated immediately after surgery, and the postoperative course was uneventful without the need for additional systemic analgesia in the recovery room. The median [interquartile range] duration of all the other surgical procedures was 82.5 [56.3–96.8] minutes.

All FLACC scores in the recovery room were <5, indicating no need for systemic analgesics during the first two postoperative hours. In those cases, where anesthesia management could be performed via neuraxial blockade and sedation without airway instrumentation, hemodynamic and respiratory parameters (heart rate, non-invasive blood pressure, and oxygen saturation) remained within normal ranges throughout the entire perioperative period.

We did not detect any side effects related to propofol. Postoperative evaluations for local infections or neurological impairment revealed no complications.

## 4. Discussion

Intestinal reconstruction is a common surgical procedure performed on infants, particularly those born prematurely [1]. The primary conditions necessitating this surgery include necrotizing enterocolitis, anorectal malformations, and intestinal atresia, often accompanied by cardiopulmonary co-morbidities, which collectively contribute to a considerable perioperative risk profile. This study examines the perioperative management of intestinal reconstruction in infants using epidural anesthesia under sedation without the need for invasive airway management. Notably, only one out of the twenty infants (5%) required endotracheal intubation due to the procedure being more extensive than initially anticipated.

The timing of intestinal reconstruction in infants depends on a balanced decision between the physical status and the expected, possible advantages of the surgical procedure. A recent retrospective study of 172 children after various intestinal diseases, as described above, indicates in 61% a growth decline, whereas 67% of infants after surgery showed a positive growth trend [12]. Thus, early intestinal reconstruction is described as outcome-essential due to absence of malnourishment and reestablishment of a physiological anatomical situs.

The median [IQR] age and weight at the time of intestinal reconstruction of infants in our study were 3.0 [2.0–6.6] months and 3.5 [3.0–6.3] kg, respectively. Half of our patients were born before the 28th gestational week. General anesthesia with invasive airway management in such a patient population is generally considered a high-risk procedure, where airway management and neurotoxicity of general anesthetic drugs require particular attention. Respiratory events during endotracheal intubation in the smallest patient population are common, and in more than 40% of intubations, two or more attempts are necessary to be successful [13]. Videolaryngoscopy may decrease the number of intubation attempts, but does not influence incidence of desaturation and bradycardia during intubation [14]. During general anesthesia, invasive airway management (endotracheal intubation or supraglottic airways) is associated with a higher risk of severe critical airway events with a relative risk of 3.36 (95% CI: 2.41–4.67) compared to the use of a face mask as an airway interface [3]. Neurotoxicity of anesthetic drugs is another concern. Although this topic remains the subject of considerable debate [15], multiple studies have identified associations between exposure to general anesthesia and neurodevelopmental outcomes [16,17,18]. The impact of general anesthesia on cerebral white matter development of the immature brain may serve as only one example in this context. The connections formed by white matter tracts between different brain regions are crucial for the integration and coordination of neural activity, playing a key role in cognitive and behavioral functions. As a result, abnormalities in white matter have been associated with impaired mobility and reduced cognitive abilities. Infants who have undergone anesthesia and surgery show a reduction in overall white matter volume compared to control subjects [19]. Thus, the quantity of general anesthetic drugs should be reduced whenever possible.

From a contemporary perspective, anesthesia management for major abdominal surgeries in infants predominantly relies on general anesthesia, where regional anesthesia serves only as a supplemental method [20]. Over the past two decades, scientific advancements have established a solid theoretical and practical foundation for the clinical application of pediatric regional anesthesia in everyday practice [21,22]. The introduction of ultrasound guidance has significantly improved the reliability of neuraxial blocks, enhancing success rates and enabling more invasive surgical procedures to be performed with epidural blockade and sedation without the need for an instrumented airway. Notably, the perioperative management of conditions such as hypertrophic pyloric stenosis or ureteric reimplantation exemplifies this approach [4,5].

Drawing on these experiences, the present study investigates these techniques involving ultrasound-guided epidural blockade combined with a well-established sedation protocol that permits spontaneous respiration and avoids the use of an instrumented airway for intestinal reconstruction surgeries. In our series, only one case (5%) necessitated invasive airway management due to the unexpected complexity of the surgical procedure and the potential risk of tracheal aspiration. This particular case involved a right hemicolectomy with ileum-to-transverse colon anastomosis, which lasted 152 min, approximately 50% longer than other procedures in the series.

Our findings suggest that epidural anesthesia without a secured airway can be both effective and safe when performed by an experienced team of anesthesiologists, surgeons, and nursing staff. Essential prerequisites for the successful implementation of this technique in daily clinical practice include maintaining a high level of vigilance throughout the perioperative period and having a comprehensive emergency plan in place for managing potential airway complications.

The findings and potential implications for the broader implementation of epidural anesthesia without a secured airway in routine clinical practice are derived from a case series. While some may criticize the absence of a randomized and comparative study for this examination, we assert that specific clinical methods can be effectively evaluated through case series research, as corroborated by the scientific literature [23,24]. Over the past years, we have investigated various clinical scenarios using case series [6,9,25]. Consequently, we maintain that the current approach of administering epidural anesthesia with sedation in spontaneously breathing patients, while avoiding invasive airway management, is adequately described by our method.

In summary, our findings support the ongoing efforts to reduce the quantity of pharmacological agents during anesthesia in our smallest patients. Regional anesthesia plays the key role in this context, and many techniques are well described with a high level of success and only minimal side effects [22]. Ultrasound-guided epidural anesthesia, performed in experienced hands, with sedation under maintenance of spontaneous breathing, is a promising anesthetic technique for intestinal reconstruction in infants. As long as the surgical procedure is based on local adhesiolysis and stoma closure, this anesthetic management can be recommended. Cooperation between surgeons and anesthesiologists and experience with ultrasound-guided neuraxial blockade are prerequisites for the successful implementation in daily clinical practice.

## 5. Conclusions

Emerging evidence supports the role of enhanced recovery protocols, opioid free approaches, and standardized perioperative care in improving outcomes in pediatric anesthesia. Performing intestinal reconstruction in infants under ultrasound-guided neuraxial anesthesia in sedated, spontaneously breathing children offers the potential to minimize airway manipulation and may reduce the need for general anesthesia agents. This study marks an initial step in demonstrating the feasibility of performing pediatric intestinal reconstruction on spontaneously breathing patients, provided the neuraxial procedure is executed with precision, a thorough understanding of potential challenges, and a multidisciplinary approach. However, additional studies with larger patient cohorts are required to determine whether this approach can be performed with sufficient success and safety.

## Figures and Tables

**Table 1:** Demographic and treatment-related data.

			*n*
Age at the time of intestinal reconstruction (months)	3.0	[2.0–6.6]	20
Gestational age	32.9	[24.5–36.9]	
Extreme preterm born (<28 weeks gestational age)			8
Very preterm born (28–32 weeks gestational age)			2
Moderate to late preterm born (28–37 weeks gestational age)			6
Weight at the time of intestinal reconstruction (kg)	3.5	[3.0–6.3]	20
Height at the time of intestinal reconstruction (cm)	47	[46.0–59.8]	20
Duration of stoma (d)	99	[60–112]	19
Duration of surgery (min)	82.5	[56.3–96.8]	20
Total volume of ropivacaine for caudal blockade (ml)	4.0	[2.7–5.2]	7
Total volume of ropivacaine for epidural anesthesia (ml)	2.6	[1.6–3.5]	13
Cases that could be managed opioid-free			19
Fentanyl (µg)	20		1
Nalbuphine (mg)	0		0

Data are median values [interquartile range]. Definitions for preterm-born infants are according to the World Health Organization (https://www.who.int/news-room/fact-sheets/detail/preterm-birth/) (Accessed date: 2 July 2025).

**Table 2:** Reasons for stoma creation (*n* = 20).

Necrotizing enterocolitis	*n* = 5	25%
Posterior sagittal anorectoplasty (PSARP)	*n* = 4	20%
Meconium ileus	*n* = 4	20%
Intestinal obstruction, volvulus	*n* = 5	25%
Focal intestinal perforation (FIP)	*n* = 2	10%

## Data Availability

The data relevant to this study cannot be made publicly available, as it contains sensitive patient information concerning a vulnerable population (infants under 1 year of age). Access to the data is restricted by the Data Access Committee of the Medical University of Vienna in accordance with Article 9 of the General Data Protection Regulation (GDPR). For inquiries or data access requests, please contact: datenclearing@meduniwien.ac.at (https://www.meduniwien.ac.at/web/en/about-us/organisation/committees/data-clearing-house/) (Access date: 2 July 2025).
